# The Contribution of AIDA (Artificial Intelligence Dystocia Algorithm) to Cesarean Section Within Robson Classification Group

**DOI:** 10.3390/jimaging11080276

**Published:** 2025-08-16

**Authors:** Antonio Malvasi, Lorenzo E. Malgieri, Michael Stark, Edoardo Di Naro, Dan Farine, Giorgio Maria Baldini, Miriam Dellino, Murat Yassa, Andrea Tinelli, Antonella Vimercati, Tommaso Difonzo

**Affiliations:** 1 Obstetrics and Gynaecology Unit, Department of Biomedical Sciences and Human Oncology, University of Bari “Aldo Moro”, 70124 Bari, Italy; antoniomalvasi@gmail.com (A.M.); edoardo.dinaro@uniba.it (E.D.N.); gbaldini97@gmail.com (G.M.B.); miriam.dellino@uniba.it (M.D.); antonella.vimercati@uniba.it (A.V.); 2 The New European Surgical Academy (NESA), 10117 Berlin, Germany; lorenzo@malgieri.org (L.E.M.); mstark@nesacademy.org (M.S.); 3 Department of Obstetrics and Gynecology, Division of Maternal-Fetal Medicine, Mount Sinai Hospital, University of Toronto, Toronto, ON M5G 1Z5, Canada; dfarine@sympatico.ca; 4 Clinic of Obstetrics and Gynecology, Şehit Prof. Dr. İlhan Varank Sancaktepe Training and Research Hospital, University of Health Sciences Turkey, 34785 İstanbul, Turkey; murat.yassa@gmail.com; 5 Clinic of Obstetrics and Gynecology, Kartal Acıbadem Hospital, 34865 İstanbul, Turkey; 6 Department of Obstetrics and Gynecology, and CERICSAL (CEntro di RIcerca Clinico SALentino), “Veris delli Ponti Hospital”, 73020 Scorrano, Italy; andreatinelli@gmail.com

**Keywords:** artificial intelligence, intrapartum ultrasound, dystocia, asynclitism, labor, cesarean section, vaginal operative delivery, malrotation, malposition, Robson classification

## Abstract

Global cesarean section (CS) rates continue to rise, with the Robson classification widely used for analysis. However, Robson Group 2A patients (nulliparous women with induced labor) show disproportionately high CS rates that cannot be fully explained by demographic factors alone. This study explored how the Artificial Intelligence Dystocia Algorithm (AIDA) could enhance the Robson system by providing detailed information on geometric dystocia, thereby facilitating better understanding of factors contributing to CS and developing more targeted reduction strategies. The authors conducted a comprehensive literature review analyzing both classification systems across multiple databases and developed a theoretical framework for integration. AIDA categorized labor cases into five classes (0–4) by analyzing four key geometric parameters measured through intrapartum ultrasound: angle of progression (AoP), asynclitism degree (AD), head–symphysis distance (HSD), and midline angle (MLA). Significant asynclitism (AD ≥ 70 mm) was strongly associated with CS regardless of other parameters, potentially explaining many “failure to progress” cases in Robson Group 2A patients. The proposed integration created a combined classification providing both population-level and individual geometric risk assessment. The integration of AIDA with the Robson classification represented a potentially valuable advancement in CS risk assessment, combining population-level stratification with individual-level geometric assessment to enable more personalized obstetric care. Future validation studies across diverse settings are needed to establish clinical utility.

## 1. Introduction

Cesarean section (CS) rates continue to increase worldwide, with variations that cannot be fully explained by clinical factors alone [1]. The Robson classification system, endorsed by the World Health Organization (WHO) and national obstetrical societies, emerged as the preferred method to monitor and compare CS rates across different settings [2]. This classification categorized women into ten groups based on obstetric characteristics, including parity, previous CS, onset of labor, fetal presentation, gestational age, and number of fetuses [3,4].

Recent analyses of CS rates using the Robson classification revealed that nulliparous women with term singleton cephalic pregnancies whose labor was induced (Robson Group 2A) had disproportionately high CS rates compared to those with spontaneous labor [5]. In Canada, for example, the cesarean rate in Group 2A was 33.5%, almost double the rate of women with spontaneous labor at 18.4%. This group made the second-largest contribution to the overall number of CSs despite comprising only 13.7% of the total obstetric population [6].

Global studies have demonstrated striking variations in CS rates within the same Robson groups across different settings. Shylla et al. revealed a stark contrast in CS rates between Robson Group 1 (0.74%) and Group 2b (69.95%) in Albania [7]. In Turkey, Birinci and Parpucu reported a concerningly high overall cesarean rate of 60.5%, with a notable rise in CSs within low-risk categories compared to WHO reference values [8]. A study from Beirut by Abdallah et al. documented an overall CS rate of 56.8%, with Groups 2 and 4 as substantial contributors [9]. These variations highlighted the need for a deeper understanding of the factors driving cesarean rates beyond the demographic and clinical characteristics captured by the Robson classification.

The high cesarean rates in Robson Class 2A highlighted a significant limitation of the classification system: while it provided a framework for comparing CS rates across populations, it did not offer insight into the specific mechanical or geometric factors that led to cesarean deliveries. This limitation was particularly relevant when considering dystocia—difficult labor due to mechanical problems—which remained one of the leading indications for primary CSs [9].

Einarsdóttir et al. provided a comprehensive analysis of CS rates specifically within Robson Groups 1 and 2a in Iceland, indicating that among the analyzed births, group 1 had a cesarean rate of 17.1%, while Group 2a contributed 11.0% to the overall CS rate of 16.4% [10]. As noted by Olerich et al., even when restricted to term, singleton, vertex pregnancies without prior CS, cesarean rates remained elevated in specific groups such as diabetic patients, with labor dystocia emerging as a major indication [11].

Recent advances in artificial intelligence, including machine learning and deep learning applications in obstetrics, have been comprehensively reviewed in the context of delivery room emergencies and labor management [12,13]. These developments encompassed various AI methodologies, from traditional machine learning to advanced deep learning approaches, providing the foundation for specialized applications like AIDA in geometric assessment of labor progression [12,14]. In this context, AIDA represented a focused application of machine learning to specific geometric parameters relevant to labor progression, building upon the broader landscape of AI applications in obstetric care.

The Artificial Intelligence Dystocia Algorithm (AIDA) utilizes four geometric parameters measured through ultrasonography: angle of progression (AoP), asynclitism degree (AD), head-symphysis distance (HSD), and midline angle (MLA). These parameters provide objective measurements of the spatial relationships between the fetus and maternal pelvis, offering potential insight into geometric dystocia. AIDA development progressed systematically from initial conceptualization through core algorithm development to focused validation studies, with recent clinical validation demonstrating 95.5% accuracy in predicting delivery outcomes for specific fetal positions [15].

The primary aim of this study was to explore how AIDA could enhance the Robson classification system, particularly for Class 2A, by providing detailed geometric information that might explain the high CS rates observed in this group. The authors hypothesized that integrating AIDA’s geometric assessment with the Robson classification would offer a more comprehensive understanding of CSs and potentially identify preventable CS within the high-risk Robson groups. This integration addressed a critical gap in current obstetric practice: the inability to objectively assess mechanical factors in labor that might lead to cesarean delivery.

## 2. Materials and Methods

### 2.1. Review of Classification Systems

This paper analyzed the methodological characteristics of two classification systems: the Robson classification system and the Artificial Intelligence Dystocia Algorithm (AIDA). The authors examined the published literature on both systems, including the original Robson classification [3], its modified version endorsed by the Society of Obstetricians and Gynecologists of Canada [4], and the recently developed AIDA framework.

Special attention was paid to publications that used the Robson classification to analyze CS rates, particularly those that identified high-risk groups such as Robson Group 2A (nulliparous women with singleton cephalic pregnancies at ≥37 weeks gestation with induced labor). The authors also reviewed the literature on geometric factors in labor dystocia, focusing on the ultrasound parameters utilized in the AIDA framework. A comprehensive literature search was conducted across multiple databases to identify studies using the Robson classification in different geographic regions and healthcare settings.

### 2.2. Robson Classification System

The Robson classification system categorized women into ten mutually exclusive groups based on obstetric characteristics as outlined in Table 1. This system was widely recognized for its simplicity, consistency, reproducibility, and adaptability. Its clinical relevance lay in its prospective categorization of women, enabling targeted implementation and evaluation of interventions within specific groups [16,17]. Unlike classification systems based on indications for CS, which often involved overlapping categories and poor reproducibility, the Robson system avoided these pitfalls with its mutually exclusive and clearly defined groups.

In some contexts, the classification itself was employed as a strategy to reduce CS rates and to assess the impact of labor induction on overall CS rates [18]. A notable strength of the system was its capacity for internal validation, as certain groups served as benchmarks. For example, Group 9—comprising women with a fetus in transverse or oblique lie—should constitute less than 1% of deliveries and exhibit a CS rate close to 100%; significant deviations from these figures might suggest issues with data accuracy or classification [19].

The Society of Obstetrics and Gynecology of Canada modified the categorization subsequently. This change subclassified women who underwent CS before labor, after labor was induced, and into spontaneous labor. The most notable subclassification was the division of Groups 2 and 4 as shown in Table 2.

The researchers noted that numerous studies worldwide had applied the Robson classification to analyze CS rates, revealing significant variations across geographic regions and healthcare settings. However, these studies also highlighted that even the modified 12-class system could not fully explain the mechanical or geometric factors that led to cesarean deliveries. This methodological limitation was particularly relevant when considering dystocia—difficult labor due to mechanical problems—which remained one of the leading indications for primary CSs.

### 2.3. AIDA Methodology and Parameters

The AIDA framework employed machine learning algorithms, specifically utilizing multiple supervised learning techniques including random forest, XGBoost, logistic regression, decision tree, support vector machine (SVM), and multi-layer perceptron (MLP) neural networks [13,16]. The algorithm incorporated a human-in-the-loop approach that prioritized physician decision-making and explainable artificial intelligence (XAI) principles, allowing systematic understanding of algorithm results. The technical implementation followed a four-step process: (1) Pearson correlation analysis to verify parameter independence, (2) supervised machine learning algorithm selection based on performance metrics, (3) decision tree analysis to establish cut-off values, and (4) delivery outcome prediction with reliability assessment.

The AIDA classification was based on four geometric parameters measured using intrapartum ultrasound.

1. Angle of progression (AoP): The angle between a line passing through the pubic symphysis and a line tangent to the fetal skull. This parameter provided objective assessment of fetal head descent through the birth canal. Lower angles indicated higher station of the fetal head and greater distance from delivery.
2. Asynclitism degree (AD): The extent of lateral tilt of the fetal head relative to the birth canal. Significant asynclitism represented a major obstacle to vaginal delivery, as it prevented the fetal head from aligning optimally with the pelvic axis. This parameter was particularly difficult to assess clinically without ultrasound.
3. Head–symphysis distance (HSD): The minimum distance between the lower margin of the pubic symphysis and the fetal parietal bone. This measurement provided an objective assessment of station beyond what could be determined by vaginal examination alone.
4. Midline angle (MLA): The angle formed by the fetal head midline and the anteroposterior diameter of the pubis. This parameter indicated the degree of rotation of the fetal head, with suboptimal angles suggesting malrotation that could impede delivery.

For each parameter, cutoff values were established to delineate normal progression (green zone), borderline values (yellow zone), and values associated with dystocia and cesarean delivery (red zone) based on previous research by Malvasi and Malgieri [13,20].

Women were then assigned to AIDA Classes 0–4 based on the number of parameters in the red or yellow zones: AIDA Class 0—all four parameters in the green zone (optimal mechanical conditions for vaginal delivery); AIDA Class 1—one parameter in the red/yellow zone, three in green (minimal mechanical impediment); AIDA Class 2—two parameters in the red/yellow zone, two in green (moderate mechanical challenge); AIDA Class 3—three parameters in the red/yellow zone, one in green (significant mechanical dystocia); AIDA Class 4—all four parameters in the red/yellow zone (severe mechanical dystocia with high likelihood of requiring cesarean delivery).

The AIDA development and validation utilized a dataset of 135 patients with 70–30 data splitting for training (95 patients) and validation (40 patients), employing five different random seed values (1, 0, 250, 500, 750) to ensure robust model validation [13]. Performance evaluation included ROC curves, AUC, accuracy, precision, recall, specificity, and F1 scores across all algorithms [13]. The current study represented a conceptual framework for integration rather than new dataset analysis, building upon the established validation of AIDA parameters. The clinical validity and reliability of these AIDA parameters were subsequently demonstrated in a prospective validation study of 66 cases with transverse fetal head positions, achieving 95.5% predictive accuracy using random forest algorithms (AIDA AND OT). This validation study confirmed the feasibility of implementing AIDA assessment using standard 3.5 MHz ultrasound equipment in routine clinical practice. Additional validation across 135 cases demonstrated clear risk stratification, with cesarean rates progressing from 0% (AIDA Class 0) to 100% (AIDA Class 4), establishing the clinical utility of geometric assessment in labor management.

### 2.4. Framework Development for Integration

While the individual AIDA components have been previously published and validated [13,14,20,21], this paper presents the first systematic framework for integrating AIDA’s geometric assessment capabilities with the globally established Robson classification system. This integration represents a novel approach to combining population-level risk stratification with individual geometric risk assessment.

A theoretical framework was developed for integrating these two classification systems, focusing on how AIDA’s geometric parameters could enhance the understanding of CSs within Robson groups, particularly in high-risk categories such as Group 2A.

The framework development process involved five key steps, as outlined below:

(a) Identifying the complementary aspects of both classification systems: The Robson system provided population-level stratification based on demographic and clinical factors, while AIDA offered individual-level assessment based on geometric parameters. Together, they addressed both the “who” (Robson) and the “why” (AIDA) of cesarean deliveries.
(b) Determining potential points of integration between Robson groups and AIDA classes: Special attention was given to high-risk Robson groups (particularly 2A) where mechanical dystocia was a common indication for cesarean delivery. The framework explored how AIDA parameters could predict or explain cesarean deliveries within these groups.
(c) Developing a conceptual model for implementation: This involved designing a practical workflow for applying both classification systems in clinical practice, including timing of assessments, integration into decision-making processes, and documentation requirements.
(d) Outlining potential benefits: The framework detailed how the integrated approach could lead to more targeted interventions, reduced unnecessary cesarean deliveries, improved risk stratification, and enhanced understanding of cesarean patterns.
(e) Describing practical implications: This involved consideration of training requirements, resource needs, and adaptation strategies for different clinical settings, from advanced tertiary centers to resource-constrained environments.

This conceptual framework was designed to provide a foundation for future research and clinical implementation, rather than presenting empirical findings from a specific dataset.

## 3. Results

### 3.1. Global Implementation of the Robson Classification System

Multiple studies worldwide have applied the Robson classification to analyze CS rates, revealing significant variations across geographic regions and healthcare settings (Table 3).

The extensive review of research spanning multiple continents demonstrated remarkable heterogeneity in CS rates both between and within countries. Researchers observed that even within identical Robson groups, CS rates exhibited striking variations—from as low as 0.74% for Group 1 in Albania [7] to 37.1% in Slovakia [24] for the same group. This substantial disparity suggested that factors beyond the demographic and clinical characteristics captured by the Robson classification significantly influenced CS decisions.

The synthesis consistently identified several Robson groups as predominant contributors to overall cesarean rates across diverse healthcare settings. Group 5 (women with previous CSs) emerged as a major contributor in numerous studies, including those from Pakistan (34.3%) [27], Nigeria (34.5%) [33], and Uganda (35.4%) [36]. Similarly, Group 2 (nulliparous women with induced labor or pre-labor CS) and Group 10 (preterm deliveries) were repeatedly identified as significant contributors. Group 2A (nulliparous women with induced labor) warranted particular attention, as this subgroup consistently demonstrated disproportionately high CS rates compared to women with spontaneous labor, despite similar demographic and clinical characteristics, as evidenced by Gu et al. [6] who found rates of 33.5% in Group 2A versus 18.4% in Group 1.

Several modifying factors influenced CS rates within Robson groups. Maternal age demonstrated a clear correlation with CS likelihood, as evidenced by Tang et al.’s findings in China [30] where rates increased from 36.1% among women aged 20–34 to 64.75% among those aged 40 and above. Medical conditions, particularly diabetes, significantly elevated CS risk, with Olerich et al. [11] documenting rates of 40.3% in gestational diabetes and 60.0% in pregestational diabetes compared to 29.7% in normoglycemic pregnancies. Additionally, non-clinical factors, including family pressure, provider convenience, financial incentives, and maternal preferences, were documented as influencing CS decisions in multiple settings, as highlighted by Nantume et al. [36].

The synthesis also revealed the potential effectiveness of quality improvement initiatives based on the Robson classification. Ji et al.’s comparative study in China [31] demonstrated that hospitals implementing strict CS reduction policies achieved significant decreases from 35% to 13.1%, while control institutions maintained high rates. Similarly, Carrillo-Aguirre et al. [25] documented successful reductions in Catalonia through targeted interventions. These findings suggested that the Robson classification provided a valuable framework for monitoring and reducing CSs, though its effectiveness was limited by its inability to account for the mechanical and geometric factors that might explain the persistent variations observed across similar populations (Table 4).

As illustrated in Table 4, eight key patterns emerged consistently across the global studies. The wide geographic variation in CS rates pointed to the influence of factors beyond clinical necessity, including healthcare system characteristics, provider practices, and cultural norms. The identification of high-risk contributors—particularly Groups 5, 2, and 10—provided valuable targets for interventions aimed at reducing unnecessary cesarean deliveries. The consistent finding of Group 2A prominence highlighted the need for specialized approaches to managing nulliparous women undergoing labor induction.

Both clinical and non-clinical factors significantly influenced cesarean decision-making. Medical conditions such as diabetes and advanced maternal age were associated with substantially higher CS rates, while non-medical factors including provider convenience and financial incentives played concerning roles in many settings. The documented success of quality improvement initiatives demonstrated the potential for change when systematic approaches were implemented, though these successes varied between public and private healthcare settings, with private facilities generally reporting higher cesarean rates.

Temporal trends revealed that despite the widespread adoption of the Robson classification as a monitoring tool, CS rates continued to rise in most settings over time. A few exceptions occurred where targeted interventions were specifically implemented and sustained, suggesting that classification alone was insufficient to reverse the global trend toward increasing CSs.

This synthesis underscored both the utility of the Robson classification as a global standard for CS monitoring and its inherent limitations in explaining the mechanical determinants of cesarean delivery, particularly in high-risk groups like nulliparous women with induced labor. The researchers concluded that while the system effectively identified which groups contributed most significantly to CS rates, it provided insufficient insight into why these cesareans occurred from a geometric perspective—a critical gap that the proposed integration with AIDA [13,20] aimed to address.

### 3.2. Complementary Nature of the Two Classification Systems

The Robson classification system and AIDA approach were designed with different but complementary purposes. The Robson classification categorized women based on obstetric characteristics to facilitate comparison of CS rates across populations and settings. In contrast, AIDA focused on objective, quantifiable geometric parameters that could directly assess the spatial relationships between the fetus and maternal pelvis during labor.

The integration of these two systems represented a potentially powerful approach to understanding CSs. While the Robson classification identified which groups of women were most likely to undergo CS, AIDA could potentially explain why these cesareans occurred from a geometric perspective, particularly in groups with high cesarean rates such as Robson Group 2A.

A fundamental limitation of the Robson classification was that it provided no insight into the mechanical or geometric factors leading to CSs, especially those performed for dystocia or “failure to progress”. This was particularly evident in the disparate CS rates observed within the same Robson groups across different settings. For example, as reported by Gu et al. [6], the Canadian cesarean rate in Group 2A was 33.5%, almost double the rate in Group 1 (18.4%), despite both groups comprising nulliparous women with term singleton cephalic pregnancies. Similarly, Záhumenský et al. [24] reported significant variability with CS rates of 37.1% in Robson Group 1 and 20.0% in Group 2b. The AIDA framework offered potential explanations for these variations through its assessment of the geometric relationship between the fetus and maternal pelvis.

### 3.3. Operational Proposal for the Integration of AIDA with the Robson Classification

A methodological framework was proposed for integrating AIDA with the Robson classification that consisted of three key components:

1. Initial Robson Classification: Women would first be classified according to standard Robson criteria, identifying high-risk groups such as 2A (nulliparous women with induced labor). This would maintain consistency with existing classification practices and facilitate continued comparison across populations and institutions.
2. AIDA Assessment: Within each Robson group, particularly those with high cesarean rates, women would undergo intrapartum ultrasound assessment of the four AIDA parameters (AoP, AD, HSD, MLA) and be assigned to AIDA Classes 0–4. For Robson Group 2A, this assessment could be performed at the time of labor induction or early in active labor to identify those at highest risk of geometric dystocia.
3. Integrated Risk Assessment: The combined Robson–AIDA classification would provide both population-level risk (Robson group) and individual geometric risk (AIDA class), allowing for more nuanced clinical decision-making. For example, a woman in Robson Group 2A with AIDA Class 0 (all parameters in green zone) might be managed differently from a woman in the same Robson group with AIDA Class 4 (all parameters in red/yellow zones).

This framework was designed to preserve the simplicity and accessibility of the Robson classification while adding the geometric precision of AIDA where it could be most beneficial. The combined classification could be expressed as “Robson 2A/AIDA 3” to indicate a nulliparous woman with induced labor who has three geometric parameters in the red/yellow zones.

### 3.4. AIDA and WHO Intrapartum Care Algorithm Working Group (ICAWG)

The WHO commissioned the development of evidence-based clinical algorithms to guide the management of both uncomplicated and complicated labors. These algorithms were designed for use by skilled health personnel providing care during labor and childbirth in any health facility, with a particular focus on low-resource settings.

In Bonet et al. [42], two evidence-based clinical algorithms for identifying and managing slow progress in first and second stages of labor were presented. These algorithms covered assessment, diagnosis, and management of labor abnormalities, focusing on low-risk pregnancies in low-resource settings. The algorithms aimed to guide healthcare providers in distinguishing normal variations from problematic situations, promoting judicious use of interventions and respectful care.

**Figure 1:**
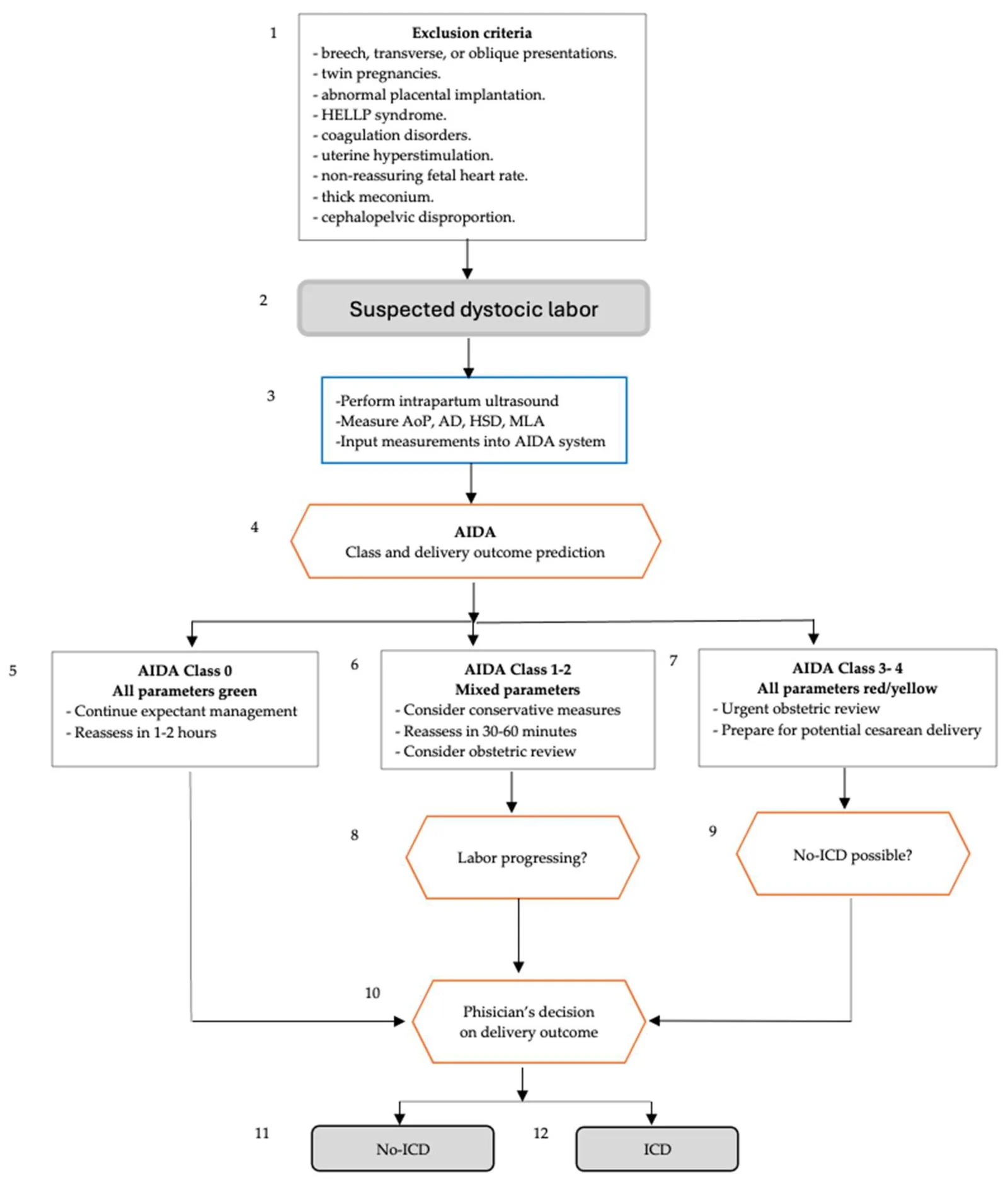
Flowchart of Suspected dystocic labor using boxes defined by the WHO Intrapartum Care Algorithm Working Group and AIDA (Artificial Intelligence Dystocia Algorithm).

In this context, the authors attempted to hypothesize an initial conceptual version of a new potential diagnosis, which they named ‘suspected dystocic labor’, designing it according to the criteria in Bonet et al. [42]. A 12-step process for assessing and managing suspected dystocic labor using the AIDA system was outlined (Figure 1), providing a structured approach incorporating both technological assessment (AIDA) and clinical judgment.

In Figure 1, two evidence-based clinical algorithms are presented for identifying and managing slow progress in the first and second stages of labor. The figure covers assessment, diagnosis, and management of labor abnormalities, focusing on low-risk pregnancies in low-resource settings. The algorithms aim to guide healthcare providers in distinguishing normal variations from problematic situations, promoting judicious use of interventions and respectful care. In the first stages of labor, the potential diagnoses include slow progress of active first stage, labor arrest, inadequate uterine activity, suspected uterine rupture, malpresentation, cephalopelvic disproportion and obstructed labor; in the second stage of labor, the potential diagnoses include slow progress of second stage, prolonged second stage, malpresentation, cephalopelvic disproportion, obstructed labor and inadequate maternal pushing efforts.

In this context, the authors attempted to hypothesize an initial conceptual version of a new potential diagnosis, which they named ‘*suspected dystocic labor*’.

The 12-step process outlined in Figure 1 was designed to guide the management of suspected dystocic labor using the AIDA system. It began by excluding high-risk conditions, and then assessed labor using intrapartum ultrasound measurements. AIDA analyzed these to predict labor class and delivery outcome. For low-risk cases (Class 0), expectant management continued. Mixed-risk cases (Classes 1–2) received conservative measures and reassessment. High-risk cases (Classes 3–4) underwent urgent obstetric review and possible cesarean preparation. The process then evaluated labor progression and the possibility of non-instrumental delivery. Finally, the physician decided the delivery method based on all the information. The algorithm ended with either non-instrumental vaginal delivery or instrumental/cesarean delivery, balancing technological assessment with clinical judgment in managing dystocic labor.

### 3.5. Potential Applications in High-Risk Robson Groups

The proposed integration had particular relevance for high-risk Robson groups, especially Group 2A. Previous studies demonstrated that this group had a cesarean rate nearly double that of women with spontaneous labor (Robson Group 1), despite similar demographic and clinical characteristics. The addition of AIDA assessment could potentially identify subgroups within 2A:

- Women with favorable geometric parameters (AIDA Class 0) who might safely continue with labor despite being in a traditionally “high-risk” Robson category: These women (approximately 25% of Group 2A based on theoretical projections) might have cesarean rates similar to those in Robson Group 1 with spontaneous labor, potentially around 10% rather than the average 33.5% for Group 2A.
- Women with moderately unfavorable parameters (AIDA Classes 1–2) who might benefit from specific interventions targeted at their particular geometric challenges: For example, women with increased asynclitism might benefit from maternal positioning strategies, while those with suboptimal angles of progression might respond to changes in oxytocin administration protocols.
- Women with highly unfavorable geometric parameters (AIDA Classes 3–4) where early intervention might be appropriate to avoid prolonged, unsuccessful labor: This group (approximately 20% of Group 2A based on theoretical projections) might have cesarean rates exceeding 50%, and early identification could prevent prolonged labor, maternal exhaustion, and fetal distress.

Several real-world applications could be envisioned for this integrated approach:

1. Pre-induction assessment: Women in Robson category 2A could undergo AIDA assessment prior to induction to better counsel them regarding their individual risk of cesarean delivery and possibly adjust the induction protocol accordingly.
2. Early labor assessment: Women in spontaneous labor (Robson Groups 1 and 3) who showed signs of slow progress could undergo AIDA assessment to determine whether geometric factors were contributing to the delay.
3. Quality improvement initiatives: Facilities with high cesarean rates in specific Robson groups could implement targeted AIDA assessment to better understand the geometric factors contributing to these rates and develop appropriate interventions.
4. Research applications: The integrated approach could facilitate research into the relationship between labor induction methods, geometric parameters, and cesarean delivery outcomes, potentially leading to more personalized induction protocols.

### 3.6. Geometric Parameters as Explanatory Factors

**Figure 2:**
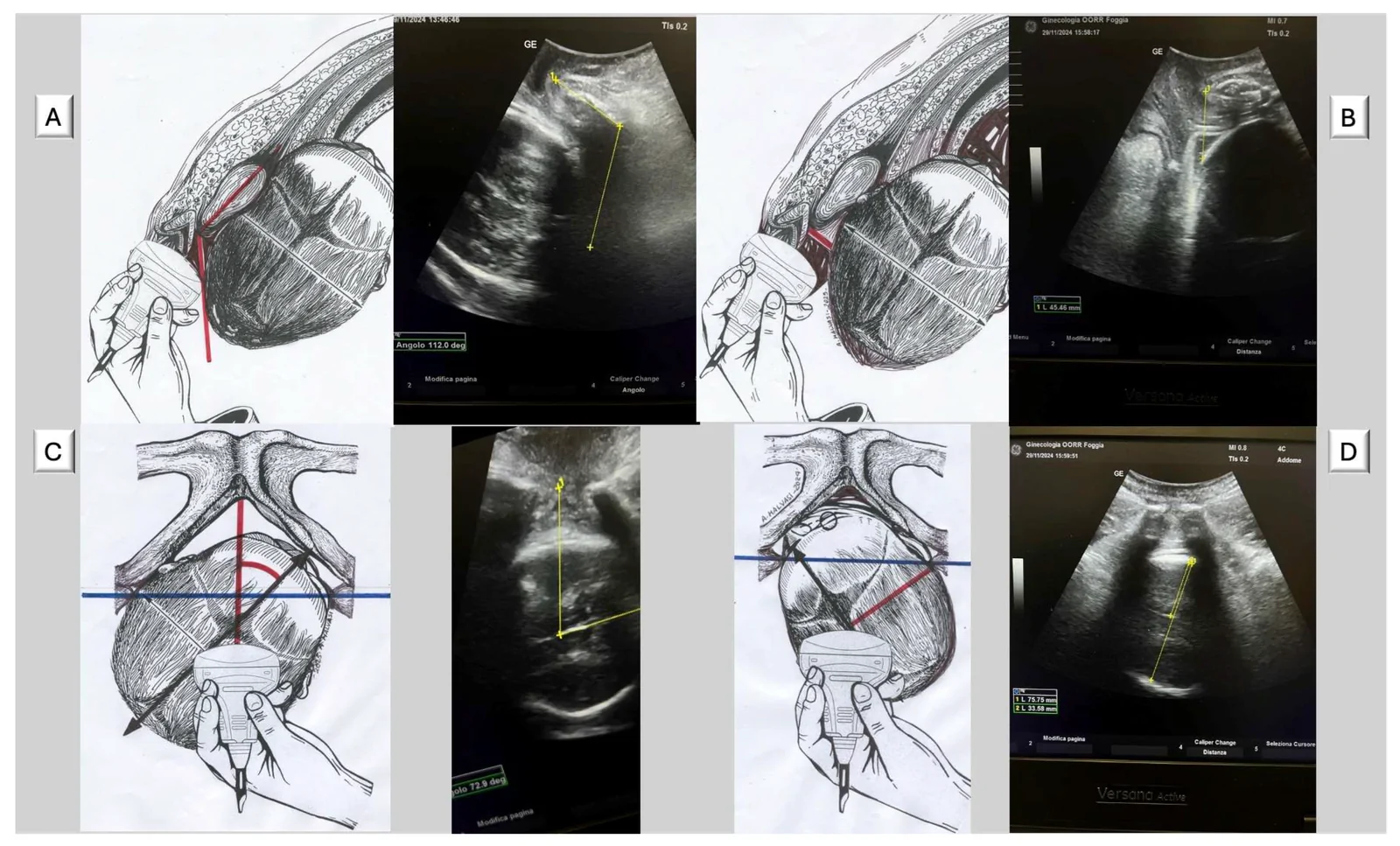
The figures illustrate the four parameters of A.I.D.A during the prolonged second stage of labor in Occiput Posterior Position. (**A**) Angle of progression (AoP): the drawing on the right (red angle) and the US photo on the left show the AoP with the fetal head in Occiput Posterior Position (O.P.P.) (yellow angle); (**B**) fetal head–symphysis distance (HSD): the drawing on the right (red line) and the US photo on the left show the HSD (yellow line) with the fetal head in Occiput Posterior Position (O.P.P.); (**C**) midline angle (MLA): the drawing on the right (red angle) and the US photo on the left (yellow angle) shows the MLA with the fetal head in the Left Occiput Posterior Position (L.O.P.P.); (**D**) asynclitism degree (AD): the drawing on the right (red line) and the US photo on the left (yellow short line) show the AD with the fetal head in the Rigth Occiput Posterior Position (R.O.P.P.). The blue transverse line indicates the biischiatic line.

The four geometric parameters measured in AIDA (Figure 2) offered potential explanations for the high cesarean rates observed in certain Robson groups. (A) Angle of Progression (AoP): This parameter can identify cases with inadequate fetal descent despite apparently normal labor progress by clinical assessment. This parameter was particularly valuable for objectively assessing station, which traditionally relied on subjective vaginal examination. Studies demonstrated that AoP < 110° was associated with an increased risk of cesarean delivery. (B) Head–Symphysis Distance (HSD): This parameter can provide objective measurement of station and descent, potentially explaining failure to progress in the second stage. HSD > 25 mm indicated that the fetal head remained high in the pelvis despite apparent progress by clinical assessment. (C) Midline Angle (MLA): This parameter can identify cases where incomplete rotation impeded vaginal delivery. MLA > 45° suggested malrotation that could significantly complicate vaginal delivery, particularly in nulliparous women with induced labor. (D). Asynclitism Degree (AD): This parameter can explain cases of labor dystocia related to unfavorable fetal head position that would not be apparent on routine clinical examination. Significant asynclitism (AD > 70 mm) prevented optimal interaction between the fetal head and maternal pelvis, impeding descent and rotation. Malvasi et al. found that severe asynclitism was strongly associated with cesarean delivery regardless of other parameters, suggesting it might be a key factor in dystocic labor.

These parameters could be particularly valuable in understanding cesarean deliveries attributed to “failure to progress” or “labor dystocia”, which remained among the most common indications for cesarean delivery, especially in nulliparous women. By objectively measuring these geometric relationships, AIDA addressed a critical gap in traditional labor assessment, which relied heavily on subjective clinical examination.

### 3.7. AIDA-Robson Integration Validation: Explaining Cesarean Rate Variations

Analysis of the complete AIDA validation cohort (*n* = 135) demonstrated the clinical value of integrating geometric assessment with Robson classification (Table 5). The data revealed striking differences in delivery outcomes between Robson Class 2 (spontaneous labor) and Class 2A (induced labor), with cesarean rates of 0% (0/57) versus 98.7% (77/78), respectively. AIDA classification explained these variations through geometric risk stratification: all women achieving vaginal delivery in the spontaneous labor group (Robson Class 2) were classified as AIDA Classes 0–1, while 75.6% of the induced labor group (Robson Class 2A) were classified as high-risk AIDA Classes 3–4.

The integration revealed that geometric factors, rather than induction per se, explained the elevated cesarean rates in Robson Class 2A. Among the four women in Class 2A who achieved vaginal delivery, three were classified as AIDA Class 1, demonstrating that favorable geometric parameters could overcome the traditionally high-risk categorization of induced nulliparous labor. Analysis of the “ICD after failure” pattern revealed that 86.4% of unsuccessful vaginal delivery attempts (19/22) occurred in AIDA Classes 2–3, while no failures occurred in AIDA Class 0. This pattern suggested that geometric assessment could identify cases where expectant management might be futile, potentially preventing prolonged labor and associated maternal morbidity. The concentration of failed attempts in intermediate AIDA classes highlighted the clinical value of objective geometric assessment in guiding delivery management decisions. The concentration of ‘ICD after failure’ cases in AIDA Classes 2–3 (19/22, 86.4%) suggested that geometric assessment could identify cases where vaginal delivery attempts were likely to be unsuccessful, potentially preventing prolonged labor and associated morbidity.

## 4. Discussion

### 4.1. Advantages of Integrating AIDA with Robson Classification

The clinical applicability of AIDA has been extensively validated in previous publications [13,20], demonstrating high accuracy rates (up to 97% with MLP algorithms) and strong associations between geometric parameters and cesarean delivery outcomes. Particularly, significant asynclitism (AD ≥ 70 mm) has been shown to be strongly associated with cesarean delivery regardless of other parameters [20], highlighting the clinical relevance of objective geometric assessment in labor management. Recent clinical validation studies established AIDA’s effectiveness in real-world practice (AIDA AND OT), achieving 95.5% accuracy in predicting delivery outcomes for transverse fetal positions and confirming the clinical utility of objective geometric assessment.

The integration of AIDA’s geometric assessment into the Robson classification system offered several potential advantages for understanding and potentially reducing cesarean deliveries, particularly in high-risk groups such as Robson 2A.

First, the combined approach addressed a fundamental limitation of the Robson classification system. While Robson effectively categorized women for comparison of cesarean rates, it provided little insight into the specific geometric factors that might lead to cesarean delivery. Multiple studies worldwide have demonstrated significant variations in cesarean rates within the same Robson groups, suggesting that factors beyond the basic demographic and clinical characteristics were at play. For example, Shylla et al. [7] reported cesarean rates of 69.95% in Robson Group 2b in Albania, while Einarsdóttir [19] et al. found rates of only 11.0% in Iceland. AIDA’s focus on objective, quantifiable geometric parameters could fill this explanatory gap, offering a spatial, three-dimensional understanding of the birth process that complemented Robson’s clinical and demographic categorization.

Second, integration could lead to more personalized risk assessment and management strategies. For example, within Robson Group 2A (nulliparous women with induced labor)—which consistently showed high cesarean rates across studies—AIDA could potentially identify subgroups at varying levels of risk. As illustrated in [13], women with favorable geometric parameters (AIDA Class 0) might have cesarean risks under 10%, despite being in a traditionally “high-risk” Robson category. Conversely, early identification of women with multiple unfavorable geometric parameters (AIDA Classes 3 and 4) might allow for more timely intervention, potentially avoiding prolonged, unsuccessful labor with associated morbidity.

Third, the combined approach could provide more specific guidance for reducing cesarean rates in high-risk Robson groups. Rather than broadly targeting all women in Robson Group 2A, interventions could be specifically directed at addressing unfavorable geometric factors identified through AIDA assessment. For example, maternal positioning techniques might be employed to address asynclitism or improve the angle of progression in women with borderline values for these parameters

Fourth, the integrated approach could enhance communication and shared decision-making between healthcare providers and women. The objective measurements provided by AIDA could facilitate clearer discussions about labor progress and the potential need for intervention, moving beyond subjective assessments of “failure to progress” to more specific explanations of mechanical challenges.

### 4.2. Potential Impact on High Cesarean Rate Groups

The potential impact of this integrated approach could be particularly significant for Robson Group 2A. This group consistently showed cesarean rates nearly double those of women with spontaneous labor (Robson Group 1), despite similar demographic and clinical characteristics. The validation data demonstrated the substantial impact of integrating geometric assessment with Robson classification, particularly for understanding the persistently high cesarean rates in Class 2A. The data revealed that geometric factors, rather than labor induction per se, explained the 98.7% cesarean rate in this population. This finding suggested that pre-induction geometric assessment could identify the small subset of women (5.1%, 4/78) with favorable parameters who might achieve vaginal delivery despite being in a traditionally high-risk category.

Analysis using the Canadian Modified Robson Classification found that Group 2A had a cesarean rate of 33.5% versus 18.4% in Group 1 [6]. This group also made the second-largest contribution to overall cesarean numbers despite comprising only 13.7% of the obstetric population.

Several factors might explain the high cesarean rates in Group 2A. Medical conditions were more prevalent in this group, with higher rates of diabetes (12.6% vs. 4.7%), hypertension/eclampsia (15.5% vs. 2.8%), and intrauterine growth restriction (5.8% vs. 1.1%) compared to Group 1 [6]. These conditions might affect labor progress and increase the likelihood of cesarean delivery. Additionally, post-date delivery was more common in Group 2A (30.1% vs. 11.2%), which could influence both the decision to induce and the success of induction.

However, these clinical factors alone might not fully explain the substantially higher cesarean rates in Group 2A. Geometric factors, as measured by AIDA, could provide additional explanatory power. Women undergoing induction might be more likely to enter labor with unfavorable fetal positioning or orientation, which could be objectively measured through AIDA parameters. For example, a high degree of asynclitism (AD) could significantly impede fetal descent and rotation, making successful vaginal delivery less likely.

The impact of reducing cesarean rates in Robson Group 2A could be substantial, not only for the individual woman’s current delivery but also for future pregnancies. Therefore, preventing a primary cesarean in a nulliparous woman could potentially prevent multiple repeat cesareans in subsequent pregnancies, with cumulative benefits for maternal morbidity, healthcare costs, and resource utilization.

Beyond Robson Group 2A, the integrated approach could also have impact on other high-risk groups, including Robson Group 1 (nulliparous women with spontaneous labor), Robson Group 5 (women with a previous cesarean and Robson Group 10 (preterm deliveries) [27,29].

### 4.3. Clinical Usability of AIDA

The clinical usability of AIDA has been demonstrated through several key features validated in previous studies [13,14,15,20].

Equipment Requirements: AIDA utilized standard transabdominal ultrasound probes (3.5 MHz) commonly available in delivery rooms, requiring no specialized equipment beyond routine obstetric ultrasound machines [13]. This ensured broad applicability across different healthcare settings without additional capital investment.

Measurement Feasibility: The four geometric parameters (AoP, AD, HSD, MLA) could be reliably measured during active labor without disrupting clinical care [13]. Assessment typically required 5–10 min and could be performed at critical decision points such as admission, labor induction, or when labor dystocia was suspected. The measurements integrated seamlessly into existing intrapartum monitoring protocols.

Clinical Integration: AIDA provided objective measurements that complemented rather than replaced clinical judgment. The five-class system (AIDA 0–4) offered clear risk stratification that clinicians could easily interpret and incorporate into decision-making [13]. Classes 0 and 4 provided particularly reliable predictions (>95% accuracy), enabling confident clinical decisions, while intermediate classes guided closer monitoring and targeted interventions.

Training Requirements: Healthcare providers familiar with basic intrapartum ultrasound could learn AIDA parameter measurement with focused training on geometric assessment techniques. The standardized protocols and decision algorithms facilitated consistent implementation across different practitioners and settings. Training modules could be integrated into existing obstetric ultrasound education programs.

Workflow Integration: AIDA assessment could be incorporated into existing labor management protocols at specific trigger points: routine assessment for high-risk Robson groups (particularly 2A), evaluation when labor dystocia was suspected, and pre-operative assessment before considering cesarean delivery. This targeted approach maximized clinical utility while minimizing workflow disruption.

### 4.4. Clinical Implications of the Integrated Approach

The integration of AIDA with the Robson classification had several important clinical implications. First, it could enhance the value of the Robson classification as a quality improvement tool. While the Robson classification helped identify which groups contributed most to cesarean rates, AIDA helped explain why these cesareans occurred from a geometric perspective, leading to more targeted improvement strategies.

Second, the combined approach could influence decision-making regarding labor induction. Current evidence suggested that elective induction in low-risk nulliparous women at 39 weeks resulted in cesarean rates similar to expectant management. However, for women with medical indications for induction, pre-induction assessment of geometric parameters might help predict the likelihood of successful vaginal delivery and guide counseling and planning [41].

Third, the integration could inform the development of more personalized labor management protocols. Rather than applying standardized approaches based solely on the Robson category, clinicians could tailor interventions to address specific geometric challenges identified through AIDA assessment. For example, women with significant asynclitism might benefit from specific maternal positioning strategies, while those with suboptimal angles of progression might require different approaches to labor augmentation.

Fourth, the integrated approach could enhance teaching and training in labor management. The objective parameters measured by AIDA could provide concrete, measurable benchmarks for assessing labor progress beyond the traditional reliance on cervical dilation and descent. This could be particularly valuable for training new clinicians in the assessment and management of labor dystocia.

Finally, the combined classification could improve documentation and communication among healthcare providers. By providing objective, quantifiable parameters alongside clinical assessments, it could facilitate clearer handovers between providers and more consistent decision-making throughout labor.

The potential clinical implications of this study were significant, as outlined below:

1. Early identification of high-risk cases: Accurate prediction of cesarean likelihood in AIDA Class 4 cases could facilitate timely intervention.
2. Reduction in unnecessary interventions: Reliable identification of low-risk cases (AIDA Class 0) could help avoid unnecessary cesarean deliveries.
3. Prevention of futile labor trials: AIDA assessment could identify cases where prolonged labor attempts were unlikely to succeed, as demonstrated by the concentration of failed vaginal delivery attempts in AIDA Classes 2–3 (86.4% of failures). This could prevent maternal exhaustion, reduce operative morbidity, and optimize timing of interventions.
4. Personalized labor management: Integration of multiple parameters allowed for more nuanced assessment of individual cases.
5. Targeted interventions: Understanding the degree of malposition could guide specific interventions.
6. Optimized timing of interventions: Earlier decision-making for cesarean delivery could potentially reduce risks associated with prolonged labor.

### 4.5. The Role of Asynclitism Degree in Geometric Dystocia

Among the four geometric parameters measured by AIDA, asynclitism degree (AD) deserved particular attention in the context of Robson classification. Asynclitism, the lateral tilting of the fetal head relative to the maternal pelvis, had been challenging to assess accurately through traditional clinical examination. AIDA’s objective measurement of AD provided valuable insights into this often-overlooked aspect of fetal positioning.

Studies of AIDA found that significant asynclitism (AD ≥ 70 mm) was strongly associated with cesarean delivery, regardless of other parameters [13,20]. This finding suggested that asynclitism might be a key geometric factor contributing to dystocic labor, particularly in Robson groups with high cesarean rates like Group 2A. Malvasi et al. [21]. described asynclitism as “an ultrasonographic rediscovery in the labor room”, highlighting its underappreciated role in labor mechanics.

The importance of asynclitism in labor mechanics had several clinical implications. It highlighted the need for assessment methods that could accurately identify and quantify this parameter. Traditional vaginal examination had limited ability to detect and quantify asynclitism, particularly in the presence of caput succedaneum or molding. Ultrasound assessment, as utilized in AIDA, provided a more objective and accurate measurement of this critical parameter.

The findings on asynclitism suggested that interventions specifically targeting this parameter might be particularly valuable in preventing some cesarean deliveries. Maternal positioning strategies, such as the use of a peanut ball or specific side-lying positions, might help correct unfavorable asynclitism and facilitate fetal descent and rotation.

### 4.6. Implementation Strategies for Different Resource Settings

The practical implementation of an integrated Robson–AIDA approach would necessarily vary across different resource settings. In high-resource settings with access to ultrasound technology and specialized training, comprehensive AIDA assessment could be offered to all women in high-risk Robson groups, particularly Group 2A.

In intermediate-resource settings, a targeted approach might be more feasible. AIDA assessment could be selectively applied to women in the highest-risk Robson groups or those with clinical signs of labor dystocia.

In low-resource settings where advanced ultrasound might not be readily available, simplified versions of the AIDA parameters could be developed. These might include basic ultrasound measurements or even clinical examination techniques that approximate the geometric relationships assessed by AIDA. For example, simplified assessment of asynclitism might be possible with basic portable ultrasound devices that are increasingly available even in resource-constrained settings.

For all settings, education and training would be crucial components of implementation. This would include training in intrapartum ultrasound techniques, interpretation of geometric parameters, and application of the integrated classification to clinical decision-making. Standardized protocols and decision support tools could facilitate consistent implementation across different practitioners and settings.

Table 6 outlines potential implementation approaches for different resource settings, acknowledging the varied availability of technology, training, and personnel.

The implementation in different settings would need to consider not only technological and training resources but also cultural and organizational factors. The acceptability of intrapartum ultrasound to women and providers, the integration into existing workflows, and the alignment with local clinical guidelines would all influence successful implementation.

### 4.7. Potential for Global Impact

Despite implementation challenges, the integrated Robson–AIDA approach had potential for global impact on cesarean delivery rates. The Robson classification has already been adopted worldwide, including in low- and middle-income countries where cesarean rates were rising rapidly. Adding AIDA assessment, even on a targeted basis for high-risk groups, could enhance understanding of cesarean patterns and guide reduction strategies.

The global impact could be assessed through several lenses:

1. Geographic breadth: As demonstrated by the numerous studies cited in this paper, the Robson classification had been applied across diverse geographic regions, from Iceland to Lebanon, China, and Ethiopia, as shown in Table 3. This suggested that an integrated approach could have truly global reach, adapting to local contexts while maintaining comparative value.
2. Healthcare system impact: The integrated approach could influence cesarean delivery rates across different healthcare systems. In public healthcare systems, where resource utilization was a key concern, the approach could help target interventions to those most likely to benefit. In private healthcare systems, where cesarean rates were often particularly high, objective geometric assessment could provide a counterbalance to non-medical factors influencing cesarean decisions.
3. Cost implications: Reducing unnecessary cesarean deliveries could have significant cost implications globally. Jamaluddine et al. [26] reported that excess CSs among Palestinian refugees during a three-year period incurred costs of up to USD 6.8 million. Across larger populations and healthcare systems, the potential cost savings could be substantial.
4. Future cesarean trajectory: Perhaps most importantly, preventing primary cesarean deliveries in nulliparous women could alter the trajectory of future cesarean rates. As noted by Jamaluddine et al. [26], rising cesarean rates among nulliparous women were identified as a key driver of future increases, as these women were more likely to undergo repeat cesareans in subsequent pregnancies.

### 4.8. Potential Mechanisms for Reducing Cesarean Rates

Understanding the geometric factors contributing to CS through the integrated Robson-AIDA approach could lead to several potential mechanisms for reducing CS rates:

1. Improved Patient Selection: By identifying women in high-risk Robson groups who nonetheless had favorable geometric parameters, clinicians might avoid unnecessary early interventions that could lead to cesarean delivery.
2. Targeted Interventions: Specific interventions could be directed at addressing unfavorable geometric factors. For example, maternal positioning techniques for asynclitism or techniques to optimize fetal head alignment during labor could be employed based on specific AIDA findings rather than general labor management protocols.
3. Enhanced Communication and Decision-Making: Objective measurements from AIDA could facilitate clearer communication about labor progress and the need for intervention, potentially increasing acceptance of appropriate cesarean delivery while avoiding unnecessary procedures. This could be particularly valuable in settings where cesarean rates were influenced by non-medical factors such as convenience or financial incentives, as noted by Nantume et al. [36].
4. Quality Improvement Initiatives: Facilities could use the combined classification to identify patterns of cesarean delivery related to specific geometric factors, potentially leading to targeted training or protocol adjustments to address these factors. For example, if high degrees of asynclitism were found to be common in women undergoing CS for “failure to progress”, specific training on managing asynclitism could be implemented.
5. Pre-induction Assessment: Women being considered for labor induction could undergo AIDA assessment to identify those with favorable geometric parameters who might have higher likelihood of successful vaginal delivery.
6. Modified Labor Management Protocols: Labor management protocols could be adapted based on AIDA findings, with different approaches for women with different geometric parameters. For example, oxytocin augmentation protocols might be modified based on the specific geometric challenges identified by AIDA assessment.

These mechanisms would need to be validated through prospective research before widespread implementation, but they offered promising avenues for addressing the persistently high cesarean rates observed in many settings worldwide.

## 5. Limitations and Future Directions

### 5.1. Limitations of the Proposed Integration

Several limitations of the proposed integration of AIDA with the Robson classification should be acknowledged. First, while the Robson classification can be applied universally in all birth settings, AIDA requires ultrasound equipment and specific training for accurate measurement of geometric parameters. This technological requirement might limit AIDA’s implementation in low-resource settings where cesarean rates are also a concern.

Second, the AIDA parameters were developed primarily in nulliparous women with prolonged second stages of labor. Their applicability to other populations, including multiparous women or those in earlier labor stages, requires further investigation. Similarly, the cutoff values for the geometric parameters might vary across different populations and settings, potentially limiting the generalizability of the AIDA classification.

Third, the integration adds complexity to an intentionally simple Robson classification system. One of the strengths of Robson is its ease of implementation and interpretation without specialized equipment or training. Adding AIDA assessment might reduce this advantage, potentially limiting adoption in some settings.

Fourth, while geometric factors are important, they represent only one aspect of the complex decision-making process surrounding cesarean delivery. The integrated approach does not fully address other important factors such as maternal preferences, provider factors, institutional policies, or non-geometric medical indications for cesarean delivery.

### 5.2. Future Research Directions

Future research to advance the integrated Robson–AIDA [40,41] approach should focus on several key areas, with active validation studies already underway to establish clinical utility and implementation frameworks. While initial clinical validation demonstrated AIDA’s effectiveness with 95.5% accuracy in transverse fetal head positions (AIDA AND OT), and subsequent validation across 135 cases confirmed clear risk stratification from 0% to 100% cesarean rates across AIDA classes, expanded validation of the integrated Robson–AIDA approach across diverse settings and populations remained essential to establish generalizability and clinical utility.

#### 5.2.1. Prospective Validation Studies

Research validating the combined approach in diverse settings and populations was essential to establish its generalizability and clinical utility. A comprehensive prospective validation study was already initiated in September 2024 (ClinicalTrials.gov: NCT06664112) to address these critical validation needs. This ongoing investigation, titled “Study of Delivery Outcomes After AIDA (Artificial Intelligence Dystocia Algorithm) Analysis”, represented the first systematic validation of the integrated classification system in real-world clinical practice.

The current validation study was designed as a prospective observational cohort investigation with an anticipated enrollment of 1000 nulliparous women at Centro di Ricerca Clinica Salentino (Italy). The study population included healthy pregnancies at first gestation, with gestational age ≥ 37 weeks, in either spontaneous or induced labor. The investigators structured the research to examine both eutocic and dystocic labor patterns, providing comprehensive insight into delivery scenarios where the integrated classification proved most beneficial.

Building upon this foundation, subsequent validation studies should include different geographical regions, healthcare systems, and patient populations. Multi-center investigations involving 5000–10,000 patients across 10–15 centers would establish generalizability across diverse populations and healthcare contexts. These expanded studies should incorporate academic medical centers, community hospitals, and private practice settings to ensure broad applicability of findings across different resource levels and practice patterns.

#### 5.2.2. Intervention Studies

Investigations examining the impact of targeted interventions based on AIDA parameters would be particularly valuable for establishing clinical utility beyond risk stratification. Studies should assess whether maternal positioning strategies designed to address unfavorable geometric factors improved outcomes in high-risk Robson groups. For example, research could evaluate whether specific positioning techniques for women with significant asynclitism (AD ≥ 70 mm) facilitated successful vaginal delivery in Robson Group 2A populations.

Additional intervention studies should examine the effectiveness of modified labor management protocols based on AIDA classifications. These investigations could assess whether oxytocin augmentation strategies tailored to specific geometric challenges improved delivery outcomes while maintaining safety profiles. The integration of AIDA assessment into existing labor management algorithms required systematic evaluation to determine optimal timing of interventions and decision-making protocols.

Pre-induction counseling studies represented another critical area for intervention research. Investigations should examine whether AIDA assessment prior to labor induction improved patient understanding of delivery risks and influenced decision-making processes. These studies could evaluate patient satisfaction, anxiety levels, and birth experience outcomes when geometric risk assessment was incorporated into informed consent processes.

#### 5.2.3. Simplified Assessment Tools

Development of simplified methods for assessing geometric parameters was crucial for enhancing practical utility across diverse healthcare settings. Research should focus on creating abbreviated protocols using basic ultrasound equipment or clinical examination techniques that approximated the geometric relationships assessed by comprehensive AIDA evaluation. These simplified approaches were particularly important for resource-constrained environments where advanced ultrasound capabilities might not be readily available.

Investigations should examine whether portable ultrasound devices could provide adequate geometric parameter assessment for clinical decision-making. Studies evaluating the accuracy and reliability of simplified asynclitism assessment using basic ultrasound technology would facilitate implementation in low-resource settings. The development of clinical examination techniques that correlated with key AIDA parameters could expand accessibility while maintaining clinical utility.

Research into automated measurement systems represented another important direction for simplification efforts. Studies should evaluate whether artificial intelligence-enhanced ultrasound systems could provide real-time geometric parameter assessment with minimal operator training requirements. These technological advances could significantly reduce implementation barriers while maintaining measurement accuracy and clinical decision-making quality.

#### 5.2.4. Educational Approaches

Research on effective training methods for intrapartum ultrasound assessment was crucial for widespread implementation of the integrated approach. Studies should evaluate simulation-based training programs, online learning modules, and competency assessment tools to determine optimal educational strategies for different practitioner groups. The development of standardized curricula for geometric parameter assessment required systematic evaluation across different training environments and practitioner experience levels.

Investigations should examine the learning curve for AIDA parameter measurement and identify factors that influenced measurement accuracy and clinical interpretation skills. Research into continuing education requirements and competency maintenance strategies would ensure consistent implementation quality over time. The effectiveness of mentorship programs and peer review processes in maintaining measurement standards represented important areas for educational research.

Studies evaluating patient education approaches were equally important for successful implementation. Research should examine how geometric risk information could be effectively communicated to patients and families, particularly in diverse cultural contexts where health literacy levels varied significantly. The development of visual aids and decision support tools for patient counseling required systematic evaluation to ensure effective risk communication and informed decision-making.

#### 5.2.5. Patient Perspectives

Investigation of patient understanding, preferences, and experiences with the integrated approach was essential to ensure that implementation respected patient autonomy and enhanced shared decision-making processes. Studies should examine patient acceptance of intrapartum ultrasound assessment and evaluate factors that influenced willingness to participate in geometric risk evaluation. Research into cultural factors affecting acceptance of AI-assisted obstetric care would inform implementation strategies across diverse populations.

Patient satisfaction studies should evaluate whether geometric risk assessment improved birth experiences and reduced anxiety related to labor management decisions. Investigations should examine whether objective risk information facilitated better understanding of clinical recommendations and improved patient confidence in care decisions. The impact of AIDA assessment on maternal perceptions of care quality and provider communication required systematic evaluation.

Research into long-term patient outcomes and satisfaction was important for establishing the broader value of the integrated approach. Studies should examine whether women who received AIDA-informed care demonstrated different patterns of future pregnancy planning, mode of delivery preferences, or birth-related anxiety in subsequent pregnancies. These investigations would provide insight into the lasting impact of geometric risk assessment on reproductive health decisions and maternal well-being.

#### 5.2.6. Current Study Outcomes and Future Directions

The ongoing NCT06664112 validation study was designed to provide crucial data for all these research directions. The comprehensive outcome assessment framework included maternal labor outcomes, neonatal complications, Apgar scores, and NICU admission requirements. This foundation would inform the development of subsequent intervention studies, educational programs, and patient communication strategies.

The study’s completion, anticipated by December 2025, will establish an evidence base for multi-center expansion and implementation science investigations. The results will inform the development of clinical practice guidelines, training curricula, and technology requirements for broader adoption of the integrated Robson–AIDA approach. This systematic research progression will ensure that implementation is supported by robust evidence while addressing the diverse needs of global obstetric practice.

Prospective Validation Studies: Research validating the combined approach in diverse settings and populations is essential to establish its generalizability and clinical utility. These studies should include different geographical regions, healthcare systems, and patient populations.

Intervention Studies: Investigations examining the impact of targeted interventions based on AIDA parameters would be particularly valuable. For example, studies could assess whether maternal positioning to address unfavorable geometric factors improves outcomes in high-risk Robson groups.

Simplified Assessment Tools: Development of simplified methods for assessing geometric parameters, potentially using basic ultrasound or even clinical examination techniques, would enhance the practical utility of the approach in diverse settings.

Educational Approaches: Research on effective training methods for intrapartum ultrasound assessment would be crucial for widespread implementation. This could include simulation-based training, online learning modules, and competency assessment tools.

Patient Perspectives: Investigation of patient understanding, preferences, and experiences with the integrated approach would ensure that implementation respects patient autonomy and enhances shared decision-making.

### 5.3. Implementation Recommendations

Based on the analysis of global Robson classification patterns and the demonstrated potential of AIDA geometric assessment, the authors recommended a systematic, evidence-based implementation strategy for integrating both classification systems. The implementation approach was designed to accommodate diverse resource settings while ensuring patient safety and clinical effectiveness across different healthcare contexts.

#### 5.3.1. Immediate Implementation (High-Resource Settings)

Healthcare institutions with comprehensive ultrasound capabilities and specialized training resources should initiate pilot implementation of the integrated Robson–AIDA approach immediately, focusing on high-risk populations where the greatest clinical benefit was anticipated.

Priority Population Focus: Pilot AIDA assessment for all Robson Group 2A patients represented the most strategic initial implementation target. This population consistently demonstrated cesarean rates nearly double those of spontaneous labor counterparts (33.5% versus 18.4%) across multiple international studies [6], suggesting substantial potential for geometric risk stratification to guide clinical decision-making. The integration would enable identification of women with favorable geometric parameters (AIDA Class 0) who might safely continue labor despite being classified in a traditionally high-risk category.

Clinical Documentation Integration: Implementation of combined Robson–AIDA classification in labor management protocols and documentation systems was essential for systematic adoption. The combined classification should be expressed as “Robson 2A/AIDA 3” to indicate both population-level risk and individual geometric assessment. Electronic medical record integration should include automated risk calculation based on ultrasound measurements, with real-time alerts for high-risk geometric patterns that might warrant clinical intervention or closer monitoring.

Comprehensive Training Programs: Establishment of comprehensive training programs for intrapartum ultrasound geometric assessment represented a critical implementation requirement. Training curricula should include Level 1 certification for basic parameter measurement (40 h program) and Level 2 certification for advanced interpretation and clinical integration (additional 20 h specialization). Annual recertification requirements would ensure maintained competency and measurement consistency across different practitioners.

Institutional Protocol Development: Development of institutional guidelines integrating both classification systems was necessary for standardized decision-making processes. These protocols should specify the timing of AIDA assessment (admission, labor induction, suspected dystocia), interpretation guidelines for each AIDA class, and escalation pathways for high-risk classifications. Quality improvement initiatives should monitor implementation metrics including protocol adherence rates, inter-observer reliability, and clinical outcome improvements.

#### 5.3.2. Targeted Implementation (Intermediate-Resource Settings)

Healthcare facilities with basic ultrasound capabilities but limited specialized training resources should adopt a selective implementation approach focusing on cases where geometric assessment would provide maximum clinical value.

Risk-Stratified Assessment Strategy: Application of AIDA assessment selectively to high-risk Robson groups with clinical signs of labor dystocia represented an efficient resource utilization strategy. Priority should be given to Robson Groups 2A and 1 (nulliparous women) where mechanical factors most commonly contributed to cesarean delivery decisions. Assessment could be triggered by prolonged labor, clinical signs of malposition, or failure to progress despite adequate contractions.

Focused Parameter Assessment: Concentration on key parameters, particularly asynclitism degree ≥ 70 mm, as primary risk indicators for clinical intervention would maximize diagnostic yield while minimizing training requirements. Studies demonstrated that significant asynclitism was strongly associated with cesarean delivery regardless of other parameters [13], suggesting this single measurement could provide substantial clinical value even in simplified implementation protocols.

Simplified Decision Algorithms: Development of simplified decision algorithms adapted to resource-constrained environments was essential for practical implementation. Paper-based decision trees could guide clinical interpretation of geometric parameters without requiring sophisticated technology infrastructure. These algorithms should provide clear action steps for each AIDA classification while maintaining integration with existing clinical protocols.

Quality Improvement Integration: Implementation of quality improvement initiatives targeting specific Robson–AIDA combinations with elevated cesarean rates would demonstrate clinical value while building institutional support. Regular audit cycles examining cesarean indications in relation to geometric parameters could identify opportunities for intervention refinement and protocol optimization.

#### 5.3.3. Graduated Implementation for Low-Resource Settings

Healthcare environments with limited ultrasound access required modified implementation strategies that maximized clinical benefit while acknowledging technological constraints.

Essential Parameter Focus: Implementation should prioritize assessment of asynclitism using basic portable ultrasound devices, as this parameter demonstrated the strongest individual association with cesarean delivery outcomes [13]. Simplified measurement protocols could enable basic geometric assessment even with limited equipment capabilities, providing valuable clinical information for high-risk cases.

Integration with WHO Algorithms: Incorporation of simplified AIDA assessment into existing WHO intrapartum care algorithms would facilitate adoption without disrupting established clinical workflows. The suspected dystocic labor algorithm (Figure 1) could be enhanced with basic geometric parameter assessment to improve diagnostic accuracy and clinical decision-making in resource-constrained settings.

Task-Sharing Implementation: Development of training programs for skilled birth attendants and midwives to perform basic geometric assessment could expand implementation reach while maintaining quality of care. Simplified competency requirements focused on recognition of significant asynclitism could provide clinical value without requiring extensive ultrasound training.

Mobile Technology Integration: Utilization of smartphone-based ultrasound systems and mobile applications for parameter calculation could overcome equipment limitations while providing standardized assessment capabilities. These technologies could enable geometric assessment in remote settings while maintaining connection to referral centers for complex cases.

#### 5.3.4. Implementation Timeline and Milestones

Phase 1 (2025–2026): Foundation Building

Based on results from the ongoing validation study (NCT06664112), initial implementation should focus on protocol refinement and training program development. High-resource settings should initiate pilot programs for Robson Group 2A patients while developing competency assessment frameworks and quality metrics.

Phase 2 (2026–2028): Scaled Implementation

Expansion to intermediate-resource settings should occur following establishment of simplified protocols and training curricula. Multi-center implementation networks should be developed to facilitate knowledge sharing and quality improvement across different healthcare contexts.

Phase 3 (2028–2030): Global Integration

Integration with international quality improvement initiatives and professional society guidelines should establish the combined classification as standard practice. Development of global training networks and technology platforms would support widespread adoption while maintaining implementation quality.

#### 5.3.5. Success Metrics and Evaluation

Primary Implementation Metrics: Success should be measured through reduction in unnecessary cesarean deliveries in target populations, particularly Robson Group 2A where 15–25% reduction represented a clinically meaningful improvement. Additional metrics included diagnostic accuracy for delivery mode prediction, protocol adherence rates, and provider confidence in clinical decision-making.

Quality Assurance Indicators: Inter-observer reliability for geometric parameter measurement (κ > 0.80), time to assessment completion, and integration with existing clinical workflows represented important process metrics. Patient satisfaction scores and birth experience outcomes provided additional measures of implementation success.

Economic Impact Assessment: Cost-effectiveness analysis comparing training and technology investments with savings from reduced cesarean deliveries would demonstrate economic value across different healthcare settings. Healthcare system burden reduction through improved labor management efficiency represented an important secondary benefit.

These implementation recommendations provided a systematic framework for adopting the integrated Robson–AIDA approach while ensuring patient safety and clinical effectiveness across diverse healthcare contexts. The phased implementation strategy acknowledged resource constraints while maximizing clinical benefit through targeted application of geometric risk assessment in obstetric care.

## 6. Conclusions

The integration of AIDA’s geometric assessment with the Robson classification system represented a methodological advancement for understanding cesarean deliveries, particularly in nulliparous women with induced labor (Robson Group 2A). While the Robson classification effectively identified which groups contributed most to cesarean rates, it provided limited insight into the geometric factors underlying these deliveries. AIDA addressed this limitation through objective measurements of spatial relationships between the fetus and maternal pelvis.

The proposed integration preserved Robson’s accessibility while adding AIDA’s geometric precision where most beneficial. This combined approach offered enhanced understanding of cesarean patterns, improved risk stratification, more personalized management strategies, and targeted interventions for cesarean reduction. Particular benefit was anticipated for Robson Group 2A, which consistently demonstrated cesarean rates nearly double those of spontaneous labor.

Despite implementation challenges related to technological requirements and training needs, the integrated approach held significant potential for advancing obstetric care.

The conceptual framework presented provided a foundation for future validation studies and implementation strategies, with the ultimate goal of improving outcomes through more precise approaches to labor management as cesarean section rates continued to rise globally. The demonstrated ability to prevent futile labor trials while identifying women who could safely achieve vaginal delivery despite being in high-risk categories represented significant potential for improving maternal outcomes and optimizing resource utilization.

## Figures and Tables

**Table 1:** The Robson classification (ten-group classification).

Groups	Obstetric Population
1	Nulliparous, single cephalic, >37 weeks, spontaneous labor
2	Nulliparous, single cephalic, >37 weeks, induced labor or pre-labor CS
3	Multiparous (excluding previous CS) single cephalic, >37 weeks, spontaneous labor
4	Multiparous (excluding previous CS) single cephalic, >37 weeks, induced labor or pre-labor CS
5	Multiparous women with at least one previous uterine scar, with single cephalic pregnancy, >37 weeks gestation
6	All nulliparous women with a single breech pregnancy
7	All multiparous women with a single breech pregnancy, including women with previous uterine scars
8	All women with multiple pregnancies, including women with previous uterine scars
9	All women with a single pregnancy with a transverse or oblique lie, including women with previous uterine scars
10	All women with a single cephalic pregnancy <37 weeks’ gestation, including women with previous scars

**Table 2:** The division of Groups 2 and 4 into subgroups according to the modified Robson classification.

**Subgroup**	**Obstetric Population**
Group 2A	Nulliparous women with singleton cephalic pregnancies at ≥37 weeks gestation with induced labor
Group 2B	Nulliparous women with singleton cephalic pregnancies at ≥37 weeks gestation who underwent cesarean delivery before labor
Group 4A	Multiparous women without previous cesarean, with singleton cephalic pregnancies at ≥37 weeks gestation with induced labor
Group 4B	Multiparous women without previous cesarean, with singleton cephalic pregnancies at ≥37 weeks gestation who underwent cesarean delivery before labor

**Table 3:** Synthesis of worldwide global Robson classification implementation studies.

Region	Country	Author [Ref]	Key Contributors (%CS)
Europe	Albania	Shylla et al. [7]	Group 1: 0.74%; Group 2B: 69.95%
	France (single center)	Jayot et al. [22]	Groups 1 and 2
	Iceland	Einarsdóttir et al. [19]	Group 1: 17.1% Group 2A: 11.0% contribution to the overall cesarean rate
	Lithuania	Kačerauskienė et al. [23]	Group 2: a reduction was observed—from 4.9% to 3.8%
	Slovakia	Záhumenský et al. [24]	Group 1: 37.1%; Group 2B: 20.0%
	Spain (Catalonia)	Carrillo-Aguirre et al. [25]	Groups 1 + 2, 3 + 4, and 10: a steady decline in CS rates from 24.3% to 22.8%
	Turkey	Birinci & Parpucu [8]	Groups 1 and 2: high overall cesarean rate of 60.5%
Middle East	Lebanon (Beirut)	Abdallah et al. [9]	Groups 2 and 4: cesarean section rate of 56.8%, with Group 2 specifically mentioned as one of the top contributors
	Palestine Refugees	Jamaluddine et al. [26]	Women with previous CS: overall rates varied significantly by setting, ranging from 22% in Gaza to 64% in Syria
Asia	Pakistan	Arshad et al. [27]	Group 5: 34.3%; Group 10: 28.5%; Group 2: 16.4% (2A: 12%)
	India	Atnurkar & Mahale [28]	Groups 2B, 4B, and 5C: no specific percentages provided for these groups
		Choudary et al. [29]	Group 2: (56 cases, 21.5%), Group 10: (56 cases, 21.5%), and Group 5: (54 cases, 20.7%)
	China	Tang et al. [30]	Groups 2, 5, and 10: 36.1% among women aged 20–34, 57.9% for those aged 35–39, and 64.75% for those aged 40 and above
		Ji et al. [31]	All groups: values dropped from approximately 35% to 13.1% by 2016
		Zhang et al. [32]	Previous CS (38.2%); maternal request (9.8%); labor dystocia (8.3%)
Africa	Nigeria	Akadri et al. [33]	Group 2: 14.0%; Group 5: 34.5%; Group 10: 12.6%
	Ethiopia	Abubeker et al. [34]	Group 2: 18.3%; Group 5: 17.1%; Group 10: 19.1%
	Sierra Leone	Arata et al. [35]	Group 1: 43.0%; Group 3:33.0%
	Uganda	Nantume et al. [36]	Group 1: 18.4%; Group 3: 13.7%; Group 5: 35.4%
Americas	Brazil	Bolognani et al. [37]	Group 1: values were higher at Hospital A (21.5%) compared to Hospital B (13.8%); Group 2B: values were 14.8% at Hospital A and 18.6% at Hospital B
	Brazil	Ramos et al. [38]	Group 1: 22.8%; Group 3: 25.7%; Group 5: 20.5%
	Brazil (Southern)	Soares et al. [39]	Group 5: 26.3%; Group 10: 17.4%
	Canada (Quebec)	Roberge et al. [40]	Previous cesarean and a term, cephalic fetus (35%), and nulliparous women with a cephalic presentation at term (30%)
	United States	Olerich et al. [11]	Cesarean delivery rates were significantly higher in patients with gestational diabetes (40.3%) and pregestational diabetes (60.0%) compared to normoglycemic pregnancies (29.7%)
Australia	Australia	Morton et al. [41]	Rising from 18.7% to 30.4% (30-year trend)

**Table 4:** Key patterns observed in the global implementation of Robson classification based on literature reviewed in Table 3.

Subgroup	Obstetric Population
Wide Geographic Variation	CS rates within the same Robson groups varied dramatically by country (e.g., Group 1: 0.74% in Albania vs. 37.1% in Slovakia).
High-Risk Contributors	Groups 5 (previous CS), 2 (nulliparous, induced/pre-labor CS), and 10 (preterm) were consistently the largest contributors across most settings.
Group 2A Prominence	Nulliparous women with induced labor (Group 2A) consistently showed disproportionately high CS rates compared to spontaneous labor counterparts.
Clinical Factors	Diabetes, maternal age, and previous CS history significantly increased CS rates within all groups.
Non-Clinical Influences	Family pressure, provider convenience, financial incentives, and maternal preferences affected CS decisions in many settings.
Quality Improvement Success	Targeted interventions based on Robson classification successfully reduced CS rates in several settings (e.g., China, Spain).
Private vs. Public Settings	Generally higher CS rates were observed in private facilities compared to public institutions.
Temporal Trends	Most settings showed increasing CS rates over time, with few exceptions where targeted interventions were implemented.

**Table 5:** Implementation strategies for different resource settings integrated AIDA–Robson classification and delivery outcomes (*n* = 135).

AIDA Class	Total Robson Class 2 (NO ICD)	Robson Class 2A (ICD)	Robson Class 2A (ICD After Failure)	Total Robson Class 2A	Total AIDA Cases
AIDA Class 0	40 (100%)	0 (0%)	0 (0%)	0 (0%)	40
AIDA Class 1	10 (71.4%)	1 (7.1%)	3 (21.4%)	4 (28.6%)	14
AIDA Class 2	4 (20%)	9 (45%)	7 (35%)	16 (80%)	20
AIDA Class 3	3 (7.9%)	26 (68.4%)	9 (23.7%)	35 (92.1%)	38
AIDA Class 4	0 (0%)	20 (87%)	3 (13%)	23 (100%)	23
Total	57 (42.2%)	56 (41.5%)	22 (16.3%)	78 (57.8%)	135

**Table 6:** Implementation strategies for different resource settings.

Resource Level	AIDA Assessment Approach	Implementation Strategy	Training Needs
High resource	Comprehensive assessment for all women in high-risk Robson groups	- Routine pre-induction assessment - Integration with electronic health records - Decision support systems - Quality improvement initiatives	- Advanced ultrasound training - AIDA parameter interpretation - Protocol development
Intermediate resource	Targeted assessment for selected high-risk cases	- Assessment for women with slow progress - Focus on highest-risk Robson groups - Paper-based decision algorithms - Selective reassessment	- Basic ultrasound training - Focused assessment protocols - Triage criteria development
Low resource	Simplified assessment using basic technology	- Abbreviated protocols - Focus on key parameters (e.g., asynclitism) - Integration with existing WHO algorithms - Task-sharing with trained midwives	- Simplified technique training - Basic interpretation skills - Integration with clinical assessment

## Data Availability

The authors of the study are custodians of the data in anonymous form, which can possibly be provided to anyone who makes a reasonable request.
